# The Valid Diagnostic Parameters in Bilateral CT Scan to Predict Unstable Syndesmotic Injury with Ankle Fracture

**DOI:** 10.3390/diagnostics10100812

**Published:** 2020-10-13

**Authors:** Si-Wook Lee, Kyung-Jae Lee, Chul Hyun Park, Hyuk-Jun Kwon, Beom-Soo Kim

**Affiliations:** 1 Department of Orthopedic Surgery, Keimyung University Dongsan Hospital, Keimyung University School of Medicine, Daegu 42601, Korea; shuk2000@naver.com (S.-W.L.); oslee@dsmc.or.kr (K.-J.L.); hyukjun0224@dsmc.or.kr (H.-J.K.); 2 Department of Orthopedic Surgery, Yeungnam University Medical Center, Hyeonchungno 170, Nam-gu, Daegu 42415, Korea; chpark77@naver.com

**Keywords:** ankle fracture, syndesmotic injury, computed tomography, tibiofibular transfixation

## Abstract

The purpose of this study is to evaluate the reasonable parameters to predict unstable syndesmotic injuries in ankle fractures. Seventy consecutive patients who underwent preoperative bilateral computed tomography (CT) scans were enrolled. Group A consisted of 20 patients intraoperatively diagnosed with syndesmotic injuries according to an intraoperative stress test and group B consisted of 50 patients who had nosyndesmotic injuries. The tibiofibular overlap (TFO) and tibiofibular clear space (TFCS) were measured using preoperative ankle radiographs. Measuring the anterior fibular distance (AFD), posterior fibular distance (PFD), anterior translation distance (AT), fibular diastasis (FD), anterior-posterior translation (APT), fibular length (FL), and surface area of syndesmosis (SAS) 1.0 and 1.5 which 1.0 cm and 1.5 cm above the tibial plafond was done via preoperative CT scan. The ratio of measurements (Injured/Intact) of the TFO, PFD, APD, and SAS 1.0 showed statistically significant differences. The researchers identified the SAS 1.0 as the most reasonable parameter to predict transfixation using receiver-operating characteristic (ROC) curve analysis. The SAS 1.0 is most valid parameter to predict syndesmotic injuries in this study and these results show that performing a bilateral CT scan on an ankle fracture may provide substantial information in measuring valid parameters.

## 1. Introduction

In unstable ankle fractures with syndesmotic injuries, an open reduction and internal fixation of fracture site and stabilization of syndesmosis are required in an effort to minimize the risk of posttraumatic arthritis [1]. Syndesmotic injuries in ankle fractures are challenging to treat in particular, not to mention relatively common in fields that require intense physical activity [2,3,4]. Measuring the tibiofibular clear space (TFCS) and the tibiofibular overlap (TFO) in a classic radiograph is the standard evaluation method [5,6,7], but they show only one side of the ankle structure. In addition, fractures around the tibia and the fibular can result in various clinical features that complicate classic radiography diagnosis. An intraoperative stress test, such as an external rotation test or hook test [8,9], can be used as more accurate dynamic test tool. However, this also has its limitations, as it requires anesthesia, preventing preoperative information from being provided.

On the other hand, computed tomography (CT) scans have recently proven effective in diagnosing fracture patterns and associated syndesmotic ankle injuries. CT scans provide a wealth of information on fracture patterns by producing clear visualizations of bones and surrounding anatomic structures. Moreover, scanning contralateral ankle images at the same time also creates a perfect “template” of a patient’s uninjured anatomy without expending too much time and effort [10,11,12]. In particular, axial CT imaging is sensitive at detecting rotational mal-reductions [13].

There are many approaches to measuring, evaluating, and predicting syndesmotic injuries. TFCS and TFO are relatively well-known measurements that can be acquired via plain radiography [5,14]. Predicting syndesmotic injuries requires the evaluation of specific parameters, namely: anterior fibular distance (AFD), posterior fibular distance (PFD), anterior translation distance (AT), fibular diastasis (FD), anterior-posterior translation (APT), and fibular length (FL) [15,16]. However, these parameters still have limits and remain inconsistent in reliably predicting syndesmotic ankle injuries [17,18,19]. The differences in reported values are the result of methodological differences in research designs, techniques used to produce the measurements, measurement errors, or differences among patient populations.

Although the CT scan is considered a valid method for detecting syndesmotic injury, there has been no substantial attempt to utilize the two-dimensional information given in CT scans. Therefore, the objective of this study was two-fold: (1) to evaluate the current parameters in predicting unstable syndesmotic injuries and (2) to validate the efficacy of the new parameters measured in bilateral CT before comparing the injured side with the intact side.

## 2. Materials and Methods

The present study reviewed 103 consecutive patients with ankle fractures who took preoperative bilateral CT scans and were operatively treated between 2008 and 2017. The researchers diagnosed these patients through physical examinations, noting their medical histories, and detailed image studies. Juveniles with incomplete growth arrest, ankle fractures with entire dislocations, a history of previous syndesmotic injury, and bilateral ankle fractures were excluded from this study. Thus, the remaining 70 patients (140 ankles) were enrolled in this retrospective study. The researchers were able to obtain the approval of the institutional review board to identify cases for this retrospective cohort study (Dongsan Hospital Institutional Review Board number: 2019-10-008; 11 October 2019).

The mean patient age at the time of injury was 39.5 years (range: 18–87 years). Meanwhile, the mean duration between the time of injury and the time of surgery was 5.74 days (range: 1–48 days). The patients were divided into two groups according to their intraoperative dynamic stress test results to compare their clinical and radiologic outcomes before and after the operation. One group (group A) consisted of 20 patients intraoperatively diagnosed with syndesmotic injuries throughout positive stress test, resulting in tibiofibular transfixation. The other group (group B) consisted of 50 patients whose intraoperative tests were negative and were consequently treated without transfixation.

### 2.1. Assessment of Syndesmotic Injury during Surgery

An unstable syndesmotic injury was confirmed under anesthesia with an external rotational stress test or hook test after the reduction and fixation of the fibular fracture. Initially, the fibular fixation was done with plate fixation, followed by fixation of the medial malleolar if there was a fracture. After the lateral and medial malleolar fracture was fixed, the posterior malleolar fracture was not fixed if there was no rotation or displacement, and if there was a posterior malleolar fracture containing more than 20% of the articular surface, fixation was performed. After fixation for these fractures was completed, a stress test was performed to find syndesmotic injury that needed fixation.

**Figure 1:**
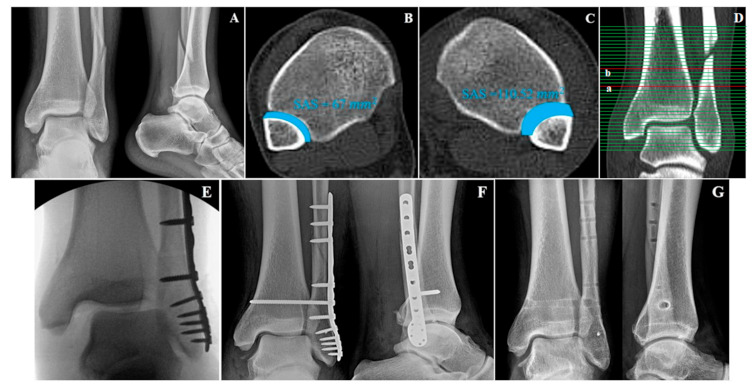
Thirty-seven-year-old male with left ankle fracture. Preoperative anterior-posterior (AP) and lateral radiographs (**A**). Bilateral axial computed tomography (CT) scan of intact (**B**) and injured ankle (**C**) with surface area of syndesmosis (SAS) measured. The ratio of his SAS (injured/intact) was 1.65. Corresponding coronal section (**D**) at level of 1.0 cm (**a**) and 1.5 cm (**b**) above the tibial plafond. Intraoperative fluoroscopic image (**E**) during stress test demonstrates possible syndesmotic injury. Immediate postoperative AP and lateral radiographs with syndesmosis transfixation included. (**F**) Last follow up of AP and lateral radiographs (**G**) with implanted devices removed without diastasis recurrence 11 months later.

During the external rotation stress test, the tibia was stabilized while the foot was neutrally flexed and externally rotated under fluoroscopy. A positive external-rotation stress test, indicated by a widened TFCS of more than 5 mm, suggested syndesmotic injury [7]. For the hook test, the surgeon pulled the lateral malleolus with a bone hook while stabilizing the tibia. Lateral fibula movement of more than 2 mm indicated a positive hook test [8]. After the fixation of the fracture, tests were performed again under fluoroscopic visualization, to clarify the syndesmotic reduction (Figure 1).

### 2.2. Clinical and Radiological Assessment

An imaging study including ankle anteroposterior radiograph and CT scan was performing before the surgery. All parameters were measured as the ratio of the intact side and the injured side, not the measured index values. This was possible because bilateral radiographs and bilateral CT were taken for this study, and this was done after obtaining patient consent. CT scans were acquired using a Siemens SOMATOM Sensation 64-slice CT scanner (Siemens, Erlangen, Germany) and a standard ankle coil with 1 mm sliced cuts. The TFCS and TFO were measured from the radiograph using a PACS viewer (INFINITT PACS; Infinitt Healthcare, Seoul, South Korea), while the AFD, PFD, AT, FD, APT, and FL [15,16,19] were measured in preoperative bilateral CT scans, 1.0 cm above the tibial plafond.

**Figure 2:**
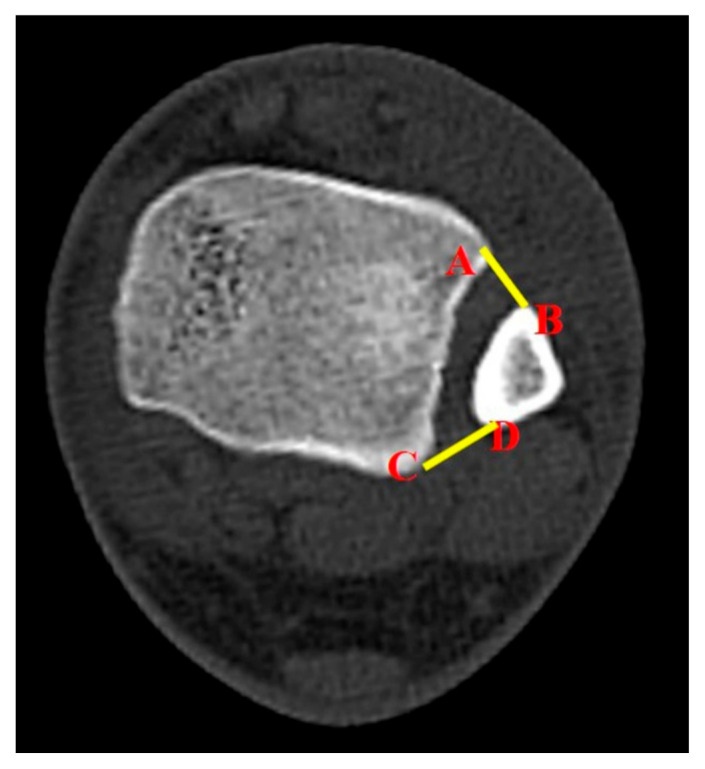
The surface area of syndesmosis, which was measured as a new parameter between four anatomic landmark points on the axial CT image; the points that link the anterior colliculus of the tibia (**A**), the most anterior aspect of the fibular (**B**), the posterior colliculus of the tibia (**C**), and the most posterior aspect of the fibular (**D**).

The study also utilized a new parameter to evaluate syndesmotic injuries. The surface area of syndesmosis (SAS) was measured 1.0 cm and 1.5 cm above the tibial plafond. From here, the researchers named them SAS 1.0 and SAS 1.5, respectively. The method for measurements of SAS 1.0 and SAS 1.5 was established by a consultative meeting made up of specialist in ankle joint trauma (SWL, CHP, BSK). The inferior border of the tibial plafond was first identified on the axial section and cross-referenced with the coronal section with scout imaging using CT scan. Each parameter (SAS 1.0 and SAS 1.5) was measured in the axial section of CT scan, which corresponds with the coronal section 1.0 cm and 1.5 cm above the tibial plafond (Figure 2). If there is a fracture line in the area to be measured at fibular site, that area was included as fracture diastasis.

### 2.3. Statistical Analysis

The researchers used a Mann-Whitney test to compare the ratio of preoperative and postoperative parameters between groups A and B. Such a comparison can confirm whether the parameters in both groups show any statistical difference. Next, the researchers performed a Shapiro-Wilk test to test the assumption of normal distribution and the homogeneity of variances. The researchers then performed a receiver-operating characteristic curve (ROC) analysis to verify the ideal parameter [20]. Furthermore, for evaluating the sensitivity and specificity, the researchers investigated with linear regression analysis using support vector machine between parameters. Statistical significance was set at a *p*-value of <0.05. Statistical analyses were performed using SPSS software 21.0 (SPSS Inc., Chicago, IL, USA).

## 3. Results

The mean age of patients mean age was 51.0 years (range: 18–78 years), with 41 males and 29 females. The affected limbs were 26 left-side ankles and 44 right-side ankles. All these cases were found to have four main causes: slip-and-fall injuries (43 cases), compression injuries from heavy objects (five cases), traffic accidents (18 cases), and falling from a height (four cases). There were no statistically significant differences between groups A and B in terms of age, sex, affected side, and body mass index (BMI) (Table 1). However, in these two groups, the fracture patterns, using the Lauge-Hansen classification system, is significantly different (Table 1).

Since patients in group B were treated without transfixation, the study put a particular focus on group A, where patients were treated with transfixation. In group A, seven out of 20 patients (35%) were treated with two transfixation materials. Two of them were with a suture-button device, while the remaining five were with cortical screws. Post-operation, six patients (30%) required the transfixation of three cortices on tibia and fibula, while the remaining 14 patients (70%) with cortical screws and a suture-button device required transfixing four cortices.

**Figure 3:**
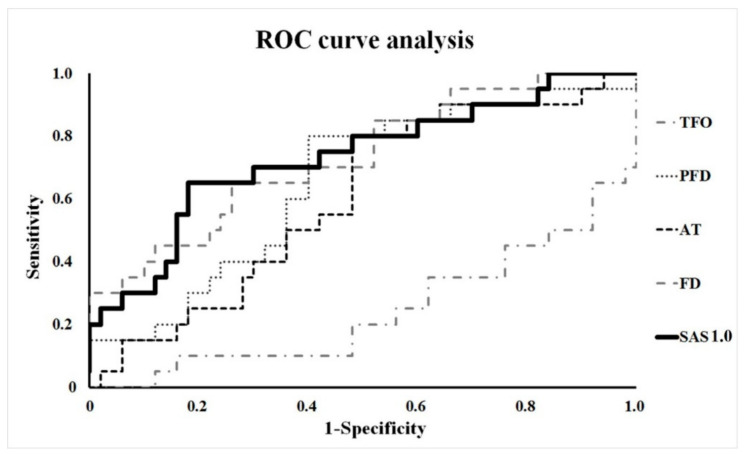
Receiver-operating characteristic (ROC) curve analysis. The result showed that the ratio of SAS 1.0 was the most reasonable, having the highest sensitivity and specificity among the other parameters of radiograph and CT (95% CI = 0.598–0.869).

Statistically significant differences were found between the ratio (injured/intact) of TFO (*p* = 0.001), PFD (*p* = 0.047), APT (*p* = 0.044), and FD (*p* = 0.003) between both groups. SAS 1.0 (*p* = 0.002) was also significant, whereas SAS 1.5 (*p* = 0.125) was confirmed as not statistically valid (Table 2). Within these proven parameters, the researchers performed a ROC curve analysis. The result showed that the ratio of SAS was the most reasonable, having the highest sensitivity and specificity among the other parameters of radiograph and CT (95% confidence interval = 0.598–0.869) (Figure 3) (Table 3). Its cut-off value is 1.56. In group A, 13 patients (65%) showed SAS 1.0 ratios higher than 1.56. In group B, six patients (12%) showed SAS 1.0 ratios higher than 1.56. In addition, TFO was the highest for the positive prediction value, and PFD and FD were the highest for the negative prediction value (Table 4). SAS 1.0 was 52% and 84% in positive prediction value and negative value, respectively, and statistically, the diagnostic significance of SAS 1.0 was similar compared to the four parameters (TFO, PFD, APT, and FD).

## 4. Discussion

The purpose of this study is to evaluate the classical radiographic parameters and newly established parameters to predict syndesmotic injuries in unstable ankle fractures using bilateral radiograph and CT scan. The results of this study revealed that TFO, PFD, APT, and SAS 1.0 were significantly reasonable parameters in judging syndesmotic injury. Among these parameters, SAS 1.0 is the most reliable reference for predicting syndesmotic injuries, and if the SAS 1.0 is 1.56 times larger than that of the intact side, there is a high possibility of syndesmotic injury. These results can predict syndesmotic stabilization prior to surgical treatment of ankle fractures and provide information on the fracture mechanism.

There are some limitations to this study. First, the surgeon who performed the diagnostic physical examination (either an external rotational stress test or a hook test) was aware of previous patient information (radiograph and CT scans), including clinical data and radiological examinations. Second, these parameters were measured only once. However, at the time of measurement, two ankle specialists and two orthopedic surgeons performed measurements through a common consensus, thus, bias has not influenced the results. Third, this study was retrospective in design, with a relatively small number of patients in subgroups. This study also focused on diagnoses and methods for assessing syndesmotic injuries, not clinical outcomes. A study with a more extended replication period, that correlates the result with the clinical and the functional outcome possible, would provide surgeons with better suggestions in deciding transfixation in syndesmotic injuries.

Despite these limitations, the present study’s findings are valuable because it included consecutive patients treated by a single surgeon at a single institution that had consistent results. In addition, studying the results of other known parameters to test and compare their validity before introducing a new valid parameter is advantageous. Furthermore, since this study was measured as a ratio through comparison with the intact side, it has the advantage of being able to obtain accurate predictions regardless of race.

The present study also sought a parameter that is more appropriate for Asian patients. There have been several studies that have reported the absolute value of the difference between the posterior and anterior tibiofibular distance in Western patients [10,21]. One study by Gardner et al. [10] stated a criterion of 2 mm for syndesmosis malreduction. Another study by Elgafy et al. [21] reported average values of 2 mm and 4 mm for the absolute distance between the tibia and fibula (anterior and posterior). However, these values do not directly apply to an Asian population. Consequently, Nault et al. [22] reported that these criteria were possibly overestimated and unreliable. Therefore, the present study’s goal was to develop a more versatile diagnostic parameter that not only includes Asian patients but also allows it to be measured and calculated with a bilateral CT scan without any additional equipment.

Syndesmotic injuries in ankle fractures that were initially observed through simple radiographs were believed to provide fair information for diagnosis [6]. The results of our study confirm that TFO is a prominent parameter for diagnosing syndesmotic injuries. However, some syndesmotic injuries in ankle fractures are occasionally concealed or overlooked in classic radiographs [23]. Specifically, tibia and fibula displacement in the anterior-posterior (AP) direction is not sufficiently observable in a radiograph’s AP view [17]. In such cases, CT scans can be an alternative diagnostic tool for presenting morphologic information and providing measured quantitative data to predict a syndesmotic ankle injury [14,24,25].

Although acquiring quantitative measurements from CT scans may vary with inspectors, it can show more insightful spatial anatomic information compared with classic radiographs. As ankle fractures can result in various disintegration patterns of anatomical structures, classic radiographs may be insufficient in providing a comprehensive understanding of the structure. These advantages leverage the CT scan as a valuable method for ankle fracture diagnosis, screening, and follow-up [10,17].

The question of which diagnostic parameter is the most accurate remains controversial [15,19]. Several studies have adopted different tools for optimal diagnosis because of the highly distinct variations in measurements and discrepancies between image and intraoperative inspections of syndesmotic injuries. In addition, the parameters suggested in prior studies were based on Western patients, whose physiques differ from most Asians [10,14,15,16,17]. Therefore, we utilized bilateral imaging to compare and minimize the differences between populations and individuals. Preoperatively diagnosing a syndesmotic injury in an ankle fracture provides various advantages to medical professionals in predicting an operation’s overall outcome, enabling advanced preparation. In this study, we found TFO, PFD, FD, APT, and SAS 1.0 as parameters with the highest potential in such preoperative diagnosis, regardless of differences among individuals.

The introduction of SAS 1.0 was a trial to utilize the two-dimensional image information in evaluating ankle fractures with syndesmotic injuries. We speculated that a two-dimensional surface area would stand out with better spatial comprehension of anatomic structures between the tibia and the fibula. The SAS 1.0 was measured 1.0 cm above the tibial plafond, which represents the deepest part of the incisura fibulae, similar to previous studies that utilized axial CT scans [9,12,13,14]. When more than one transfixation between tibia and fibula is performed, the area 15 mm above it is commonly transfixed [9,25]. Therefore, by analyzing the level of measurement, the SAS 1.5 was measured 1.5 cm above the tibial plafond and resulted in no better validity than the SAS measured 1.0 cm above the tibial plafond. This study confirmed that the SAS 1.0 ratio could be a useful diagnostic tool for syndesmotic injuries.

Although SAS 1.0 showed the most statistical validity among all radiological parameters, only 13 patients out of 20 in group A had a SAS 1.0 ratio higher than the cut off value of 1.56. The SAS 1.0 ratio of six out of 50 patients from group B was also higher than 1.56, but transfixation was still not performed. These results demonstrate a fair number of syndesmotic injuries concealed by radiological diagnosis. Therefore, these injuries require an intraoperative stress test to confirm. Although numerous trials were using preoperative radiologic imaging, which allows surgeons to predict syndesmotic injuries, the present study indicates that an intraoperative stress test has better potential. Future studies with larger cohorts, valid parameters, and different measurement levels, and studies that include volumetric measurements in a three-dimensional context, may provide more information.

## 5. Conclusions

The results of this study showed that TFO, PFD, APT, and SAS 1.0 were significantly reasonable parameters in judging syndesmotic injury. In particular, this study demonstrated that performing preoperative bilateral CT scans and measuring the SAS 1.0 to compare with the uninjured side are useful and reliable diagnostic methods for predicting syndesmotic injuries in ankle fractures.

## Figures and Tables

**Table 1:** Patient demographics.

	Group A * (*n* = 20)	Group B ** (*n* = 50)	*p*-Value
Sex (male:female)	14:06	3:23	0.156
Mean age (year)	46.4	52.9	0.116
Side of injury (Right:Left)	11:9	33:17	0.775
Body mass index (kg/m^2^)	24.05	24.83	0.662
Duration since trauma till operation (days)	7.57	5.02	0.788
Mechanism of injury			0.064
Traffic accident	8	10	
Slip-and-fall	7	36	
Fall from a height	3	1	
Compression	2	3	
Lauge-Hansen Classification of fracture			<0.001
Supination adduction	0	4	
Supination external rotation	1	42	
Pronation abduction	1	2	
Pronation external rotation	18	2	

* Group A, patients who had suspected syndesmotic injury under intraoperative stress test; ** Group B, patients who were suspected not to have syndesmotic injury under intraoperative stress test.

**Table 2:** The ratio of measurements of ankle with syndesmotic injury and intact ankle.

Radiologic Reference Line	Group	Mean Ratio (Injured/Intact)	SD	*p*-Value
TFO	A *	0.57	0.27	0.001
	B **	0.87	0.39	
TFCS	A	1.51	0.87	0.131
	B	1.14	0.53	
AFD	A	1.32	0.47	0.11
	B	1.15	0.51	
PFD	A	1.25	0.52	0.047
	B	1.04	0.29	
AT	A	1.22	0.44	0.178
	B	1.13	0.61	
FD	A	1.78	0.99	0.003
	B	1.12	0.38	
APT	A	1.16	0.27	0.044
	B	1.01	0.18	
Fibular length	A	1.01	0.11	0.104
	B	1.09	0.2	
SAS 1.0	A	1.98	0.98	0.002
	B	1.41	0.33	
SAS 1.5	A	1.47	0.58	0.125
	B	1.37	0.22	

* Group A, patients who had suspected syndesmotic injury under intraoperative stress test; ** Group B, patients who were suspected not to have syndesmotic injury under intraoperative stress test; SD, standard deviation; TFO, tibiofibular overlap; TFCS, tibiofibular clear space; AFD, anterior fibular distance; PFD, posterior fibular distance; AT, anterior translation distance; FD, fibular diastasis; APT, anterior-posterior translation; SAS 1.0, surface area of syndesmosis that was 1.0 cm above the tibial plafond; SAS 1.5, surface area of syndesmosis that was 1.5 cm above the tibial plafond.

**Table 3:** The receiver-operating characteristic curve analysis of the ratio of parameters (injured/intact).

Radiologic Parameter	Area	Standard Error	Significance Probability	Approximate 95% CI	
				Lower Limit	Upper Limit
TFO	0.245	0.068	0.001	0.112	0.378
PFD	0.653	0.071	0.047	0.513	0.793
APT	0.655	0.078	0.044	0.501	0.809
FD	0.732	0.067	0.003	0.600	0.863
SAS 1.0	0.734	0.069	0.002	0.598	0.869

TFO: tibiofibular overlap; PFD: posterior fibular distance; APT: anterior-posterior translation; FD fibular diastasis: SAS 1.0; surface area of syndesmosis that was 1.0 cm above the tibial plafond.

**Table 4:** The sensitivity, specificity, and positive and negative predictive values of ratio of parameters.

Radiologic Parameter	Sensitivity	Specificity	Positive Predictive Value (%)	Negative Predictive Value (%)
TFO	0.920	0.500	71	82
PFD	0.800	0.600	44	88
APT	0.650	0.580	38	81
FD	0.850	0.460	39	88
SAS 1.0	0.650	0.820	52	84

TFO: tibiofibular overlap; PFD: posterior fibular distance; APT: anterior-posterior translation; FD fibular diastasis: SAS 1.0; surface area of syndesmosis that was 1.0 cm above the tibial plafond.
